# Grading of Chinese Cantonese Sausage Using Hyperspectral Imaging Combined with Chemometric Methods

**DOI:** 10.3390/s17081706

**Published:** 2017-07-25

**Authors:** Aiping Gong, Susu Zhu, Yong He, Chu Zhang

**Affiliations:** 1 College of Mechanical and Electrical Engineering, Shenzhen Institute of Information Technology, Shenzhen 518172, China; gongap@sziit.edu.cn; 2 College of Biosystems Engineering and Food Science, Zhejiang University, Hangzhou 310058, China; sszhu@zju.edu.cn

**Keywords:** near-infrared hyperspectral imaging, Chinese Cantonese sausage, random forest, quality grading

## Abstract

Fast and accurate grading of Chinese Cantonese sausage is an important concern for customers, organizations, and the industry. Hyperspectral imaging in the spectral range of 874–1734 nm, combined with chemometric methods, was applied to grade Chinese Cantonese sausage. Three grades of intact and sliced Cantonese sausages were studied, including the top, first, and second grades. Support vector machine (SVM) and random forests (RF) techniques were used to build two different models. Second derivative spectra and RF were applied to select optimal wavelengths. The optimal wavelengths were the same for intact and sliced sausages when selected from second derivative spectra, while the optimal wavelengths for intact and sliced sausages selected using RF were quite similar. The SVM and RF models, using full spectra and the optimal wavelengths, obtained acceptable results for intact and sliced sausages. Both models for intact sausages performed better than those for sliced sausages, with a classification accuracy of the calibration and prediction set of over 90%. The overall results indicated that hyperspectral imaging combined with chemometric methods could be used to grade Chinese Cantonese sausages, with intact sausages being better suited for grading. This study will help to develop fast and accurate online grading of Cantonese sausages, as well as other sausages.

## 1. Introduction

Sausage is a meat product with world-wide popularity. Cantonese sausage is a kind of traditional Chinese-style semi-dry sausage that occupies the majority of the sausage market in China, due to its exotic texture, flavors, and taste [1]. Sausage quality is greatly influenced by the processing parameters, such as ingredients used [2,3,4,5].

Different quality grades of Cantonese sausage are produced to meet the demands of consumers and significant price differences exist between these different grades. The price differences have caused the emergence of counterfeit products and the intentional mislabeling of grades. As the sausage industry draws big business, organizations and industries involved in production are trying to ensure the quality and pricing of Cantonese sausage. Fast and accurate certification of Cantonese sausage grades is demanded by consumers, management, and quality control departments. Accurate grading of Cantonese sausage provides accurate indications for consumption and pricing, and can help to protect the consumer’s rights, and certify the quality to satisfy the quality assurance department for a legitimate sausage producer. 

Hyperspectral imaging is a contactless technology integrating two techniques, such as near-infrared spectroscopy and imaging techniques. Each pixel within a hyperspectral image contains a full spectrum, and each wavelength has a grayscale image. Hyperspectral imaging provides comprehensive internal and external information of the samples. Studies have reported using hyperspectral imaging as an effective technique to determine physicochemical and sensory features of meat and meat products [6,7,8,9,10].

Studies have been conducted to detect the lean and fat proportions in meat [11]. However, sausage is complex due to the mixture of different proportions of lean and fat meat, as well as other ingredients. It is important to explore the feasibility of using hyperspectral imaging to grade meats with a complex composition like sausage. 

Near-infrared spectroscopy has been used to detect the quality of sausage [12,13,14,15,16]. However, the use of hyperspectral imaging for sausage grading and quality detection has not been reported. Generally, intact sausages are sold in markets. With the development of prepared food, sliced sausages were provided in markets for consumer convenience. The objective of this study was to grade intact and sliced Chinese Cantonese sausage using hyperspectral imaging combined with chemometric methods. The specific objectives were to explore the feasibility of using hyperspectral imaging to grade Chinese Cantonese sausage, to develop and compare the grading models for intact and sliced sausages, and to identify important wavelengths for Chinese Cantonese sausage grading.

## 2. Materials and Methods 

### 2.1. Sample Preparation

Three different grades of the well-known brand of Chinese Cantonese sausage ‘Xishangxi’ were collected from the local supermarket, including the top grade, first-grade, and second-grade. The sausages were vacuum-packed. The major difference between the three grades of sausage were the proportion of lean and fat meat. The top grade had the leanest meat and the least fat, and the second-grade had the least lean meat and the most fat. 

**Figure 1:**
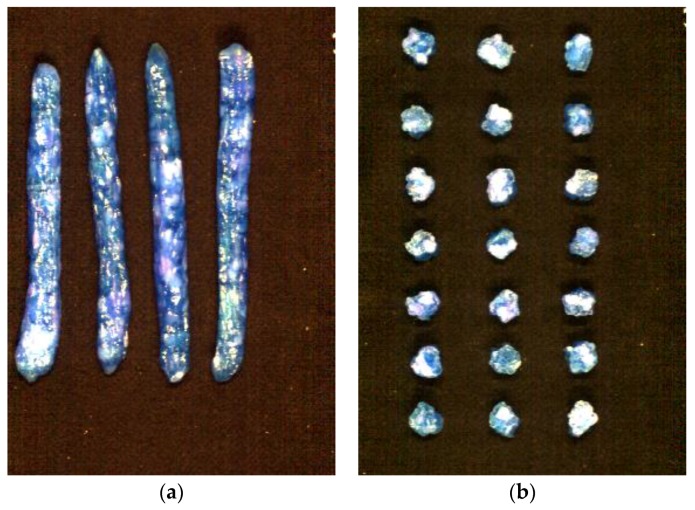
Pseudo images of (**a**) intact and (**b**) sliced sausages (generated from images at 1000, 1200, and 1400 nm).

Fifty intact sausages of each grade were collected. After hyperspectral images were taken of the intact sausages, three slices were cut from the middle part of the intact sausage with a slice thickness of 1 cm. In total, 150 slices of each grade were collected. Then, hyperspectral images were taken of the sliced sausages. Figure 1 shows sample images of the intact and sliced sausages.

### 2.2. Image Acquisition and Correction

The hyperspectral images of intact and sliced sausage were taken in an assembled hyperspectral imaging system. The main component of the system is an imaging spectrograph (ImSpector N17E; Spectral Imaging Ltd., Oulu, Finland) covering the spectral range of 874–1734 nm and a 320 by 256 CCD camera (Xeva 992; Xenics Infrared Solutions, Leuven, Belgium) with a camera lens (OLES22; Specim, Spectral Imaging Ltd.). This system takes images with the line-scan method. 

A white reference image and a dark reference image were acquired to remove dark current and correct light intensity, respectively. The white reference image was acquired using a piece of white Teflon with nearly 99% reflectance, and the dark reference image was acquired by turning off the light source and covering the camera lens completely with its opaque cap. Image correction was conducted using the following equation

(1) $$Icor=\frac{Iraw-Idark}{Iwhite-Idark}$$

where *Icor* is the corrected image, *Iwhite* is the white reference image, and *Idark* is the dark reference image.

### 2.3. Spectral Data Extraction

To extract spectral data, regions of interest (ROI) were defined. For intact sausages, the entire sample region in the hyperspectral image was defined as the ROI; for sliced sausages, the entire sample region of sliced sausage was defined as the ROI. The pixel-wise spectra contained obvious noises, and wavelet transform with Daubechies 6 wavelet function with decomposition level 3 was applied to the pixel-wise spectra within each ROI [17]. The average spectrum of all pixels within each ROI was extracted.

### 2.4. Data Analysis Methods

#### 2.4.1. Evaluation Methods

Support vector machine (SVM) is a widely-used pattern recognition method. SVM maps inseparable samples into higher dimensional space to find a linear classifier to classify samples. A hyperplane (or a set of hyperplanes) is constructed to classify samples. The hyperplanes which maximize the distance between the nearest samples of different classes are selected. Kernel functions are essential to map the original data into the higher dimension space. The radial basis function (RBF) is a widely-used kernel function in SVM for spectral data analysis. A penalty coefficient (C) and kernel width (γ) of RBF-based SVM is determined by a grid-search procedure [18].

Random forests (RF) is an ensemble learning method. RF contains a batch of individual decision trees for classification and regression. In RF, the tree bagging procedure is used for training, and during training, the samples and features are randomly split for each tree. The number of trees and number of nodes on each tree need to be determined to establish a RF. RF can handle large amounts of data efficiently, and RF shows a high tolerance for noise and outliers [19].

#### 2.4.2. Optimal Wavelength Selection

Hyperspectral images generate a large amount of data, which can be difficult to handle. The spectral data suffer from collinearity and redundancy. Optimal wavelength selection aims to select a few wavelengths that most contribute to sample features from the original spectra while maintaining model performance. Optimal wavelength selection can also reduce the amount of data, reduce the number of computation tasks, and simplify the model. In this study, two different variable selection methods were used for optimal wavelength selection.

Second derivative spectra are mainly used for spectral preprocessing as they maintain the spectral features, highlight the spectral peaks, and suppress the background information. Spectral peaks with large differences within the second derivative spectra can be selected as optimal wavelengths [20].

As introduced in Section 2.4.1, RF is an efficient modelling method for classification and regression. One of the characteristics of RF is the ability to evaluate the importance of each variable. A variable selection procedure by RF is used according to the procedure introduced by [19]. First, a RF model is built to obtain an initial ranking of importance for each variable. Second, variables with lower importance are eliminated, and the first and the second steps are repeated until the number of remaining variables equals a predefined number-N. The remaining N variables are ranked in decreasing order according to their importance. Third, a RF model is built on the new variable subsets from one to N variables. The out of bag (OOB) error of each model is calculated. The variables in the RF model with the lowest OOB error values are selected as optimal variables.

#### 2.4.3. Model Performance Evaluation and Software

The performance of the models was evaluated by classification accuracy of the calibration and prediction sets. The classification accuracy is defined as the percentage of correctly classified samples in all samples. The chemometric methods were all conducted on MATLAB R2014b (The Math Works, Natick, MA, USA).

## 3. Results and Discussion

### 3.1. Spectral Profiles

**Figure 2:**
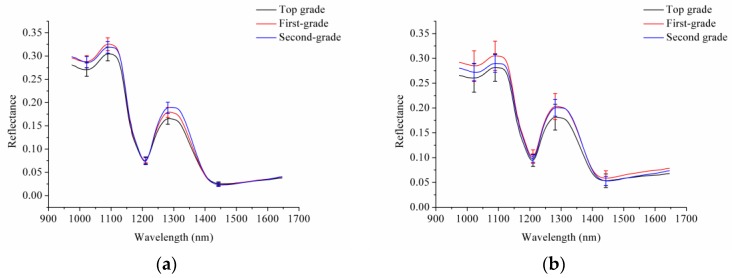
(**a**) Average spectra with SD of the wavelengths at the spectral peaks and valleys for three grades of intact sausages, and (**b**) average spectra with SD of the wavelengths at the spectral peaks and valleys for three grades of sliced sausages. The bold lines refer to the average spectra, and the vertical lines of the corresponding color indicate the SD of the wavelengths at the spectral peaks and valleys.

To reduce the noise caused by the hyperspectral imaging system and the environment, only spectra in the range of 975.01–1645.82 nm, for a total of 200 wavelength variables, were studied. The average spectra, along with the standard deviation (SD) of the wavelengths at the spectral peaks and valleys for different grades of intact and sliced sausages, are shown in Figure 2.

As shown in Figure 2a, overlapping was observed between the average spectra with SD of the wavelengths at the spectral peaks and valleys for different grades of intact sausages. Slight differences were also observed between different grades of intact sausages. As shown in Figure 2b, overlapping was also observed between the average spectra with SD of the wavelengths at the spectral peaks and valleys for different grades of sliced sausages. Slight differences were observed among different grades of sliced sausages. The spectra of the sliced sausages showed greater variations than the spectra of intact sausages. 

### 3.2. PCA Analysis

**Figure 3:**
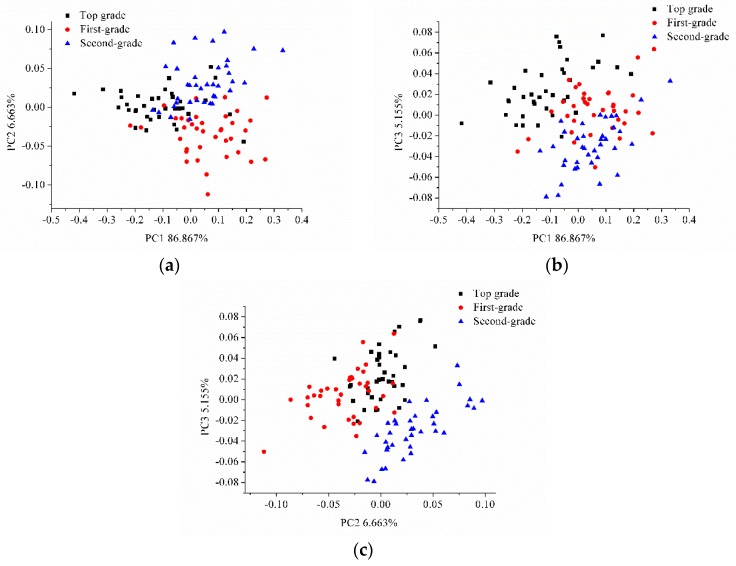
Score scatter plots of (**a**) PC1 vs. PC2, (**b**) PC1 vs. PC3, and (**c**) PC2 vs. PC3 for intact sausages.

**Figure 4:**
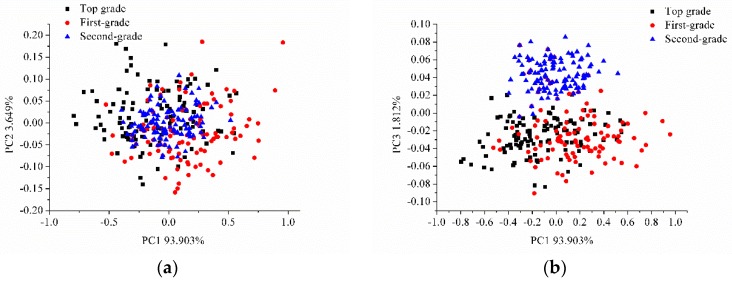

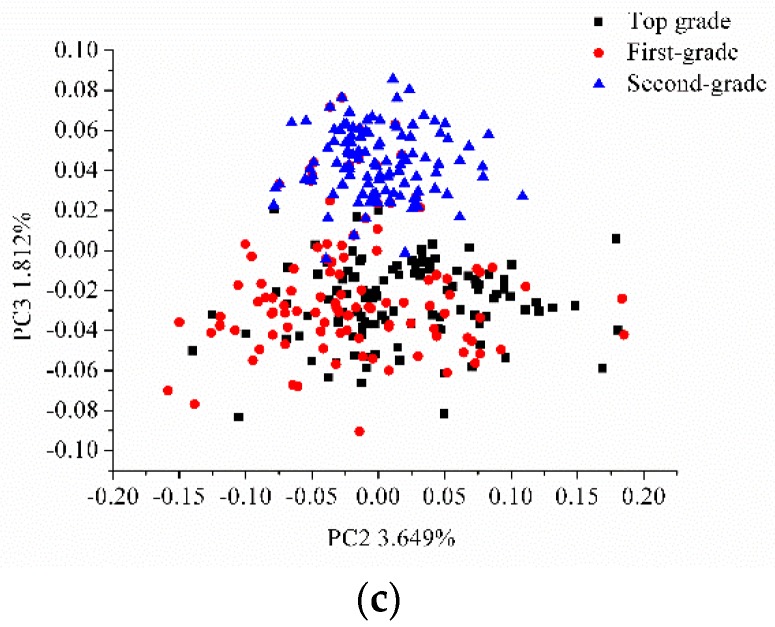
Score scatter plots of (**a**) PC1 vs. PC2, (**b**) PC1 vs. PC3, and (**c**) PC2 vs. PC3 for sliced sausages.

PCA was conducted on the average spectra of the three grades of intact and sliced sausages for qualitative analysis. PCA analysis was also conducted on the hyperspectral images of the intact and sliced sausages. The score scatter plots of PC1 and PC2, PC1 and PC3, and PC2 and PC3 for intact sausages are shown in Figure 3. The score scatter plots of PC1 and PC2, PC1 and PC3, and PC2 and PC3 for sliced sausages are shown in Figure 4.

Slight overlapping could be found in Figure 3, between the different grades of sausages, however the three grades of intact sausages could be classified. Greater overlapping of the sliced sausages of the three grades was observed in Figure 4a, though the sliced sausages of the three grades separated well and could be classified in Figure 4b,c. The results of PCA analysis indicated the feasibility of using hyperspectral imaging to grade Cantonese sausages.

### 3.3. Classification Models Using Full Spectra 

The intact and sliced samples were divided into calibration and prediction sets by the Kennard–Stone algorithm. Of the intact samples, 37 of each grade were divided into the calibration set, and the remaining 13 samples from each grade were divided into the prediction set. Of the sliced sausages, 111 of each grade were divided into the calibration set, and the remaining 39 samples of each grade were divided into the prediction set. The top grade, first-grade, and second-grade sausages were assigned the category value of 1, 2, and 3, respectively. SVM and RF models were built using the full spectra. SVM conducts a grid-search procedure to search for the optimal combination of SVM parameters (C, γ). The number of trees in RF models was from 50 to 500, with intervals of 50. The number of features selected for each node in a tree was investigated from 10 to 100, with intervals of 10. The results of the SVM and RF models for intact and sliced sausages are shown in Table 1.

For intact sausages, the classification results by the SVM and RF models were satisfactory, with over 90% accuracy in the classification results for calibration and prediction. For sliced sausages, the classification results from SVM and RF models were less accurate. As shown in Table 1, the first-grade intact and sliced sausages could be misclassified as the top grade or the second-grade using RF. This phenomenon was more obvious with the sliced sausages. The reason for this might be that sausages are mixtures of lean meat, fat meat, and other ingredients. The intact sausages have a more uniform distribution of lean meat, fat meat, and other ingredients in each grade. However, the distribution of lean meat, fat meat, and other ingredients in the sliced sausages of each grade might not be uniform, resulting in poor grading performance. The results indicate that hyperspectral imaging combined with other methods could be used to grade Cantonese sausages.

### 3.4. Optimal Wavelength Selection

**Figure 5:**
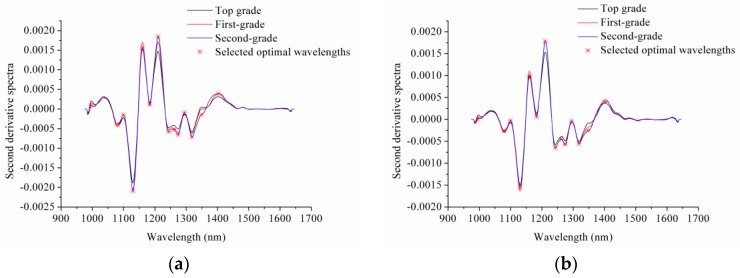
Optimal wavelengths selected by second derivative spectra of (**a**) intact sausages and (**b**) sliced sausages.

Second derivative preprocessing was performed on the average spectra of the sausages of the three grades. The peaks in the second derivative spectra with large differences were selected as optimal wavelengths. The optimal wavelengths selected by the second derivative spectra are shown in Figure 5 and Table 2. As shown in Figure 5 and Table 2, the second derivative spectra of the intact and sliced sausages were similar, and the selected optimal wavelengths were the same for the intact and sliced sausages. The reason for this might be that the compositions of intact and sliced sausages of each grade were the same, with the differences being due to sampling surfaces. 

**Figure 6:**
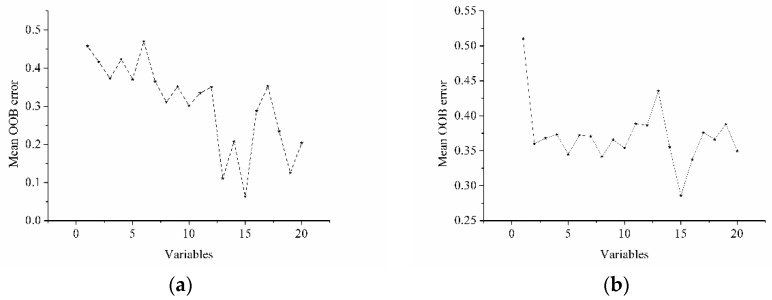
Mean OOB error values of RF models using different wavelength variables: (**a**) intact sausages; (**b**) sliced sausages.

RF was also applied to select optimal wavelengths. First, 100 RF models were built using full spectra with the RF parameters presented in Table 1. Second, the mean importance of each wavelength in the 100 RF models was calculated and followed by the elimination of the 20 wavelengths with lowest mean importance. Third, 100 RF models were built using the remaining wavelengths with the RF parameters. Fourth, the second and third steps were repeated until only 20 wavelengths remained. Finally, the remaining 20 wavelengths were ranked in decreasing order, and 100 RF models were built using the first *k* wavelengths (*k* = 1 to 20), and the mean OOB error was calculated. The wavelengths with the lowest OOB error were selected as optimal variables. The wavelengths and OOB error plots are presented in Figure 6. Fifteen optimal wavelengths were selected for the intact and sliced sausages.

For intact sausages, the remaining 20 wavelength variables with the greatest mean importance were selected, including 1291, 1338, 1328, 1278, 1315, 1311, 1318, 1325, 1348, 1321, 1079, 1342, 1301, 1332, 1069, 1288, 1072, 1436, 1284, and 1086 nm. For sliced sausages, the remaining 20 wavelength variables with the greatest mean importance were selected, including 1072, 1318, 1082, 1069, 1062, 1328, 1066, 1056, 1335, 1315, 1338, 1321, 1076, 1089, 1332, 1079, 1288, 1038, 1308, and 1086 nm. As shown in Figure 5, the first 15 variables were selected as optimal wavelengths. 

The remaining 20 wavelengths for the intact and sliced sausages were similar. RF for variable selection depended on a random selection procedure. However, the similarity of the optimal wavelengths selected by RF indicated RF was effective for optimal wavelength selection. 

The selected optimal wavelength of 995 nm was attributed to the second overtone of N–H [21]; the selected optimal wavelengths between 1000 and 1100 nm (1056, 1062, 1066, 1069, 1072, 2076, 1082, 1089, and 1099 nm) were attributed to the second overtone of N–H stretching [22]; 1160 nm was attributed to the second overtone of the C–H stretching band [23]; 1210 nm was attributed to the second overtone of C–H [24]; the selected optimal wavelengths between 1254 and 1348 nm were attributed to a combination of the first overtone of Amide B with the fundamental Amide II and III vibrations [25]; and the selected optimal wavelength near 1400 nm (1402 nm) may be attributed to O–H bonds [26].

### 3.5. SVM and RF Models Using Optimal Wavelengths

To evaluate the performance of the selected optimal wavelengths, SVM and RF models were built for the intact and sliced sausages. The results are shown in Table 3. The performance of each model for the intact sausages was satisfactory with classification accuracies of the calibration and prediction set of over 90%. Both models for sliced sausages obtained slightly less accurate yet acceptable results. The results indicated that the optimal wavelengths selected through both second derivative spectra and RF could be used for Cantonese sausage grading.

Comparing the results in Table 1 and Table 3, both models using optimal wavelengths obtained similar results as those using full spectra. However, the number of variables for each model was reduced from 200 to 14 and 15, resulting in a significant reduction in the amount of data (over 92.5%). Hyperspectral imaging generates a large amount of data, resulting in significant data processing tasks, which is a drawback when applying the technique to real-world problems. The results in this study show the effectiveness of optimal wavelength selection by different methods. The use of optimal wavelengths has the potential to develop an online multi-spectral imaging system using the selected optimal wavelengths for food quality and safety control at lower costs.

In this study, intact and sliced sausages were used to explore the feasibility of using hyperspectral imaging to grade Cantonese sausages. The results indicated that although intact and sliced sausages could both be used for Cantonese sausage grading, intact sausages were better suited for grading with this method. The main difference among the different grades of Cantonese sausages (brand: Xishangxi) was the proportion of lean and fat meat. The mixtures of lean and fat meat in sausages were not perfectly homogeneous, the intact sausages could present more detailed information about the mixture of lean and fat meat. 

Although intact and sliced sausages showed differences in model performance, the optimal wavelengths selected by second spectra for intact and sliced sausages were the same, and the optimal wavelengths selected by RF for intact and sliced sausages were quite similar. The reason for this may be due to the fact that the ingredients used to produce Cantonese sausages are the same.

## 4. Conclusions

Hyperspectral images were used to grade Chinese Cantonese sausages, and spectral information was extracted from the intact and sliced sausages. The SVM and RF models that were built using full spectra obtained acceptable results, and both models for intact sausages performed better than those for sliced sausages. The results indicated the feasibility of using hyperspectral imaging to grade Cantonese sausages. RF and second derivative spectra were used to select optimal wavelengths for Cantonese sausage grading. Optimal wavelengths selected through second derivative spectra for intact and sliced sausages were the same, and optimal wavelengths selected by RF for intact and sliced sausages were quite similar. SVM and RF models, using the selected optimal wavelengths, obtained acceptable results, and both models using optimal wavelengths for intact sausages performed better than those for sliced sausages. The classification accuracy of both models using full spectra and the optimal wavelengths for intact sausages was over 90%. The similarity of the optimal wavelengths selected for intact and sliced sausages, and the performances of the models using optimal wavelengths, indicate the effectiveness of optimal wavelength selection through RF and second derivative spectra. The overall results indicate that hyperspectral imaging combined with chemometric methods for optimal wavelength selection could be used to grade Cantonese sausages. The results in this study will help to provide a new, fast, accurate, and non-destructive online alternative for Cantonese sausage grading, which would benefit consumers, organizations, and industry.

## Figures and Tables

**Table 1:** Grading results of intact and sliced sausages by SVM and RF models.

		Parameters *		Calibration Set				Prediction Set			
				1 ^a^	2 ^a^	3 ^a^	Total (%)	1	2	3	Total (%)
Sliced	SVM	84.4485, 9.1896	1	100	8	3		36	3	0	
			2	12	77	22		9	23	7	
			3	4	0	107		0	0	39	
							85.29				83.76
	RF	50, 60	1	111	0	0		31	8	0	
			2	1	104	6		10	20	9	
			3	0	10	101		0	4	35	
							94.89				73.50
Intact	SVM	256, 3.0314	1	35	2	0		13	0	0	
			2	1	35	1		0	13	0	
			3	0	0	37		0	0	13	
							96.40				100.00
	RF	50, 50	1	37	0	0		13	0	0	
			2	0	37	0		2	11	0	
			3	0	0	37		0	0	13	
							100.00				94.87

* Parameters indicate the model parameters of each model, i.e., (C, γ) for SVM, and the number of trees in the forest and features for each node on a tree. The parameters were identical for the methods in different tables in this manuscript; ^a^ top grade, first-grade, and second-grade of sausages are represented by 1, 2, and 3, respectively.

**Table 2:** Selected optimal wavelengths by second derivative spectra and RF for intact sausages and sliced sausages.

Methods	Intact		Sliced	
	Number	Wavelengths (nm)	Number	Wavelengths (nm)
Second derivative spectra	14	995, 1079, 1099, 1130, 1160, 1183, 1210, 1244, 1261, 1274, 1294, 1318, 1348, 1402	14	995, 1079, 1099, 1130, 1160, 1183, 1210, 1244, 1261, 1274, 1294, 1318, 1348, 1402
RF	15	1291, 1338, 1328, 1278, 1315, 1311, 1318, 1325, 1348, 1321, 1079, 1342, 1301, 1332, 1069	15	1072, 1318, 1082, 1069, 1062, 1328, 1066, 1056, 1335, 1315, 1338, 1321, 1076, 1089, 1332

**Table 3:** Results of the RF and SVM models using selected optimal wavelengths.

		SVM (%)			RF (%)		
		Parameters	Calibration Set	Prediction Set	Parameters	Calibration Set	Prediction Set
Intact	Second derivative spectra	256, 9.1896	95.50	100.00	50, 50	100.00	94.87
	RF	147.0334, 147.0334	90.09	94.87	50, 50	100.00	92.31
Sliced	Second derivative spectra	256, 48.5029	82.28	87.18	50, 60	94.59	78.63
	RF	256, 147.0334	80.78	85.47	50, 60	94.89	76.07
